# Author Correction: Genetic variants of calcium and vitamin D metabolism in kidney stone disease

**DOI:** 10.1038/s41467-022-30920-5

**Published:** 2022-05-30

**Authors:** Sarah A. Howles, Akira Wiberg, Michelle Goldsworthy, Asha L. Bayliss, Anna K. Gluck, Michael Ng, Emily Grout, Chizu Tanikawa, Yoichiro Kamatani, Chikashi Terao, Atsushi Takahashi, Michiaki Kubo, Koichi Matsuda, Rajesh V. Thakker, Benjamin W. Turney, Dominic Furniss

**Affiliations:** 1 Nuffield Department of Surgical Sciences, University of Oxford, Oxford, UK; 2 Academic Endocrine Unit, Radcliffe Department of Medicine, University of Oxford, Oxford, UK; 3 Nuffield Department of Orthopaedics, Rheumatology and Musculoskeletal Sciences, University of Oxford, Oxford, UK; 4 Laboratory of Genome Technology, Human Genome Centre, University of Tokyo, Tokyo, Japan; 5 RIKEN Centre for Integrative Medical Sciences, Yokohama, Kanagawa Japan; 6 Laboratory of Clinical Genome Sequencing, Department of Computational Biology and Medical Sciences, University of Tokyo, Tokyo, Japan

**Keywords:** Calcium signalling, Genome-wide association studies, Calcium and vitamin D, Renal calculi

Correction to: *Nature Communications* 10.1038/s41467-019-13145-x, published online 15 November 2019

The original version of this Article contained an error in the first paragraph of the Association analyses subsection of the Methods, which incorrectly read ‘Three covariates were used in the association study: genetic sex, age, and the genotyping platform (to account for array effects).’ The correct version replaces this sentence with ‘Two covariates were used in the association study: genetic sex, and the genotyping platform (to account for array effects).’ This has been corrected in both the PDF and HTML versions of the Article.

