# Supplementary material for: Genetic variants of calcium and vitamin D metabolism in kidney stone disease
Source: Nat Commun. 2019 Nov 15;10:5175. doi: 10.1038/s41467-019-13145-x (PMC6858460; doi:10.1038/s41467-019-13145-x)
Supplement: Supplementary file 1 — Supplementary Information [file 41467_2019_13145_MOESM1_ESM.pdf]

Supplementary Information

This appendix has been provided by the authors to give readers additional information about their work

Supplement to: Genetic variants of calcium and vitamin D metabolism in kidney stone disease. Howles et al.

1 Supplementary Tables 3

Supplementary Table 1: Inclusion criteria for identification of stone forming individuals in UK Biobank population. 3

Supplementary Table 2: Exclusions for UK Biobank population.......... 4

Supplementary Table 3. SNPs significantly associated with kidney stone disease in UK Biobank population 5

Supplementary Table 4. Heterogeneity in effects of index SNPs between UK Biobank GWAS and Japanese GWAS. 6

Supplementary Table 5. Genes implicated in FUMA positional mapping. 7

Supplementary Table 6: Comparison of genotypic models for rs1716707. 8

Supplementary Table 7: Genotype-phenotype correlations in cohort of kidney stone formers at rs1170174 9

Supplementary Table 8: UK Biobank Study Population. 9

Supplementary Table 9: Japanese Study Population. 10

Supplementary Table 10: Genomic inflation in the GWA studies. 10

Supplementary Table 11: Primers 11

2 Supplementary Figures 12

Supplementary Figure 1: Regional plots of all GWAS-associated loci in the UK-Japanese meta-analysis. 12

Supplementary Figure 2: Gene-property analysis in MAGMA. 18

Supplementary Figure 3: Gene-based enrichment analysis in FUMA. 19

Supplementary Figure 4: Intake of medications in validation cohort participants shown across genotypes. 20

Supplementary Figure 5: CaSR-mediated responses following DGKD knockdown in HEK-CaSR and HEK-CaSR-NFAT cells. 21

1 Supplementary Tables

Supplementary Table 1: Inclusion criteria for identification of stone forming individuals in UK Biobank population. ICD-10, OPCS and self-reported operation codes used to define renal stone cases in the association analysis. The number of individuals with each diagnostic code within the post-QC UK Biobank dataset is shown.

ICD-10 Codes		N	OPCS codes		N	Self-reported operation code	N
N20.0	Calculus of kidney	3,064	M06.1	Open removal of calculus from kidney	24	1197 Percutaneous/open kidney stone surgery/lithotripsy	2,549
N20.1	Calculus of ureter	2,288	M09.1	Endoscopic ultrasound fragmentation of calculus of kidney	64		
N20.2	Calculus of kidney with calculus of ureter	327	M09.2	Endoscopic electrohydraulic shock wave fragmentation of calculus of kidney	28		
N20.9	Urinary calculus, unspecified	111	M09.3	Endoscopic laser fragmentation of calculus of kidney	211		
N23	Unspecified Renal Colic	1,843	M09.4	Endoscopic extraction of calculus of kidney	192		
			M09.8	Other specified therapeutic endoscopic operations on calculus of kidney,	18		
			M09.9	Unspecified therapeutic endoscopic operations on calculus of kidney	15		
			M14.1	Extracorporeal shock wave lithotripsy of calculus of kidney	1,434		
			M14.8	Other specified extracorporeal fragmentation of calculus of kidney	66		
			M14.9	Unspecified extracorporeal fragmentation of calculus of kidney	73		
			M16.4	Percutaneous nephrolithotomy	108		
			M27.1	Ureteroscopic laser fragmentation of calculus of ureter	545		
			M27.2	Ureteroscopic fragmentation of calculus of ureter	208		
			M27.3	Ureteroscopic extraction of calculus of ureter	347		
			M28.1	Endoscopic laser fragmentation of calculus of ureter	76		
			M28.2	Endoscopic fragmentation of calculus of ureter	50		
			M28.3	Endoscopic extraction of calculus of ureter	123		
			M28.4	Endoscopic catheter drainage of calculus of ureter	3		
			M28.8	Other specified other endoscopic removal of calculus from ureter	3		
			M28.9	Unspecified other endoscopic removal of calculus from ureter	9		
			M31.1	Extracorporeal shock wave lithotripsy of calculus of ureter	588		
			M31.8	Other specified extracorporeal fragmentation of calculus of ureter	1		
			M31.9	Unspecified extracorporeal fragmentation of calculus of ureter	9		
			M26.1	Nephroscopic laser fragmentation of calculus of ureter	5		
			M26.2	Nephroscopic fragmentation of calculus of ureter NEC	4		
			M26.3	Nephroscopic extraction of calculus of ureter	7		
			M28.5	Endoscopic drainage of calculus of ureter by dilation of ureter	2		
			M28.8	Other specified other endoscopic removal of calculus from ureter	3		

Supplementary Table 2: Exclusions for UK Biobank population. ICD-10 and OPCS codes used to exclude individuals from the association analysis. The number of individuals with each diagnostic code within the post-QC UK Biobank dataset is shown.

ICD-10 Codes		N	OPCS codes		N
E26.81	Bartter syndrome	0	M39.1	Open removal of calculus from bladder	19
E72.0	Disorders of amino acid transport	9	M44.2	Endoscopic extraction of calculus of bladder	122
E21.0	Hyperparathyroidism	476	M67.4	Endoscopic removal of calculus from prostate	5
E21.1	Hyperparathyroidism	46	M75.8	Open extraction of calculus from urethra	9
E21.2	Hyperparathyroidism	12	G27.1	Gastric bypass surgery	9
E21.3	Hyperparathyroidism	336	G27.2	Gastric bypass surgery	7
Q61.5	Medullary sponge kidney	29	G27.3	Gastric bypass surgery	1
N25.8	Type 1 renal tubular acidosis	52	G27.4	Gastric bypass surgery	17
K50	Inflammatory bowel disease	1,681	G27.5	Gastric bypass surgery	28
K51	Inflammatory bowel disease	3,097	G27.8	Gastric bypass surgery	8
K91.2	Postsurgical malabsorption	30	G28.1	Gastric bypass surgery	20
Q62	Congential obstructive defects of the renal pelvis and malfomations of the ureter	57	G28.2	Gastric bypass surgery	20
			G28.3	Gastric bypass surgery	30
E83.31	Hereditrary hypophosphatemic rickets with hypercalciruria and nephrolithiasis, osteoporosis and hypophosphatemia	0	G28.4	Gastric bypass surgery	3
			G28.5	Gastric bypass surgery	66
E83.42	Familial hypomagnesemia with hypercalciuria and nephrocalcinosis and Familial hypomagnesemia with hypercalciuria and nephrocalcinosis with ocular abnormalities	0	G28.8	Gastric bypass surgery	14
			G28.9	Gastric bypass surgery	35
E74.8	Oxaluria and oxalosis	5	G31.1	Gastric bypass surgery	9
N21.0	Calculus in bladder	691	G31.2	Gastric bypass surgery	3
N21.1	Calculus in urethra	50	G31.3	Gastric bypass surgery	0
N21.8	Other lower urinary tract calculus	31	G31.4	Gastric bypass surgery	0
N21.9	Calculus of the lower urinary tract	7	G31.8	Gastric bypass surgery	0
			G31.9	Gastric bypass surgery	0
			G31.0	Gastric bypass surgery	0
			G32.1	Gastric bypass surgery	67
			G32.2	Gastric bypass surgery	2
			G32.3	Gastric bypass surgery	2
			G32.4	Gastric bypass surgery	0
			G32.8	Gastric bypass surgery	13
			G32.9	Gastric bypass surgery	1
			G32.0	Gastric bypass surgery	0
			G33.1	Gastric bypass surgery	249
			G33.2	Gastric bypass surgery	11
			G33.3	Gastric bypass surgery	0
			G33.6	Gastric bypass surgery	3
			G33.8	Gastric bypass surgery	0
			G33.9	Gastric bypass surgery	2
			G33.0	Gastric bypass surgery	1

Supplementary Table 3. SNPs significantly associated with kidney stone disease in UK Biobank population

Chromosome	Position[a]	rsID	Effect Allele	Non-Effect Allele	EAF[b]	INFO score	OR (95% CI)	P	Candidate Gene
1	21836934	rs6703976	T	C	0.11	0.998	1.18 (1.12-1.25)	3.7×10^{-9}	*ALPL*
1	21893344	rs1256332	A	C	0.16	0.995	1.17 (1.12-1.23)	6.2×10^{-11}	*ALPL*
2	234296444	rs838717	G	A	0.43	0.995	1.11 (1.07-1.15)	1.6×10^{-8}	*DGKD*
5	17679999	rs10051765	C	T	0.33	0.995	1.16 (1.11-1.20)	7.8×10^{-15}	*SLC34A1*
6	160619918	rs28495851	C	A	0.03	0.989	1.35 (1.22-1.50)	4.7×10^{-9}	*SLC22A2*
7	27653207	rs7790498	A	G	0.30	0.999	1.13 (1.08-1.17)	2.9×10^{-10}	*HIBADH*
13	42688211	rs1170174	A	G	0.18	0.988	1.16 (1.11-1.22)	4.5×10^{-11}	*DGKH*
16	20392332	rs77924615	A	G	0.20	0.98	1.13 (1.09-1.18)	1.8×10^{-8}	*UMOD*
20	52732362	rs17216707	T	C	0.81	0.961	1.17 (1.12-1.22)	9.9×10^{-12}	*CYP24A1*
21	37818871	rs2776288	A	G	0.63	0.988	1.18 (1.14-1.22)	5.7×10^{-19}	*CLDN14*
22	23410918	rs13054904	A	T	0.26	0.999	1.15 (1.11-1.20)	3.3×10^{-12}	*BCR*

[a]Based on NCBI Genome Build 37 (hg19).[b]The effect allele frequency in kidney stone formers. Two independent signals were identified at the *ALPL* locus.

Supplementary Table 4. Heterogeneity in effects of index SNPs between UK Biobank GWAS and Japanese GWAS. The Q-statistics and corresponding p-values are shown for each of the 20 index SNPs that were genome-wide significant in the trans-ethnic meta-analysis. Out of 20 loci, 3 loci demonstrated heterogeneity between the two GWAS at $p < 0.05$ (bold italic).

Chromosome	SNP ID	Q statistic	Q statistic p-value
1	***rs10917002***	***4.179***	***0.041***
2	rs780093	3.853	0.050
2	rs13003198	0.127	0.721
4	rs1481012	0.079	0.779
5	rs56235845	0.346	0.556
6	rs1155347	0.659	0.417
6	rs77648599	0.840	0.359
7	rs12539707	0.234	0.628
7	rs12666466	0.506	0.477
11	rs4529910	3.133	0.077
13	***rs1037271***	***8.465***	***0.004***
15	rs578595	0.419	0.518
16	rs77924615	0.513	0.474
16	rs889299	0.104	0.747
17	***rs1010269***	***5.750***	***0.016***
17	rs4793434	0.003	0.957
19	rs3760702	1.697	0.193
20	rs17216707	1.846	0.174
21	rs12626330	1.092	0.296
22	rs13054904	0.893	0.345

Supplementary Table 5. Genes implicated in FUMA positional mapping.

Gene	Symbol[a]	Entrez ID	Chromosome	Start[b]	End
ENSG00000162551	*ALPL*	249	1	21835858	21904905
ENSG00000115211	*EIF2B4*	8890	2	27587219	27593353
ENSG00000115234	*SNX17*	9784	2	27593389	27599995
ENSG00000163795	*ZNF513*	130557	2	27600098	27603657
ENSG00000115241	*PPM1G*	5496	2	27604061	27632554
ENSG00000084734	*GCKR*	2646	2	27719709	27746554
ENSG00000221843	*C2orf16*	84226	2	27799389	27805588
ENSG00000243943	*ZNF512*	84450	2	27805897	27858041
ENSG00000176714	*CCDC121*	79635	2	27848506	27851879
ENSG00000198522	*GPN1*	11321	2	27851114	27874375
ENSG00000119760	*SUPT7L*	9913	2	27873679	27886676
ENSG00000163798	*SLC4A1AP*	22950	2	27886338	27917840
ENSG00000205334	*AC074091.13 (LINC01460)*	100129995	2	27928653	27938599
ENSG00000243147	*MRPL33*	9553	2	27994584	28210954
ENSG00000171174	*RBKS*	64080	2	28004231	28113965
ENSG00000158019	*BRE*	9577	2	28112808	28561768
ENSG00000130561	*SAG*	6295	2	234216462	234255701
ENSG00000077044	*DGKD*	8527	2	234263153	234380750
ENSG00000085982	*USP40*	55230	2	234384166	234475428
ENSG00000118762	*PKD2*	5311	4	88928820	88998929
ENSG00000118777	*ABCG2*	9429	4	89011416	89152474
ENSG00000165671	*NSD1*	64324	5	176560026	176727216
ENSG00000169228	*RAB24*	53917	5	176728199	176730745
ENSG00000213347	*MXD3*	83463	5	176728462	176739758
ENSG00000169230	*PRELID1*	27166	5	176730775	176733960
ENSG00000169223	*LMAN2*	10960	5	176758563	176778853
ENSG00000169220	*RGS14*	10636	5	176784838	176799602
ENSG00000131183	*SLC34A1*	6569	5	176806236	176825849
ENSG00000196570	*PFN3*	345456	5	176827108	176827637
ENSG00000131187	*F12*	2161	5	176829141	176836577
ENSG00000198055	*GRK6*	2870	5	176830205	176869902
ENSG00000164626	*KCNK5*	8645	6	39156749	39197226
ENSG00000175003	*SLC22A1*	6580	6	160542821	160579750
ENSG00000112499	*SLC22A2*	6582	6	160592093	160698670
ENSG00000106049	*HIBADH*	11112	7	27565061	27702614
ENSG00000254959	*INMT-FAM188B*	100526825	7	30791753	30931696
ENSG00000106125	*FAM188B*	84182	7	30811033	30932002
ENSG00000240583	*AQP1*	358	7	30893010	30965131
ENSG00000110777	*POU2AF1*	5450	11	111222977	111326355
ENSG00000102780	*DGKH*	160851	13	42614176	42830714
ENSG00000166415	*WDR72*	256764	15	53805938	54055075
ENSG00000169344	*UMOD*	7369	16	20344374	20367623
ENSG00000169340	*PDILT*	204474	16	20370492	20416059
ENSG00000168447	*SCNN1B*	6338	16	23289552	23392620
ENSG00000141376	*BCAS3*	54828	17	58754814	59470199
ENSG00000123143	*PKN1*	5585	19	14543865	14582679
ENSG00000160951	*PTGER1*	5731	19	14583278	14586174
ENSG00000123159	*GIPC1*	10755	19	14588572	14606944
ENSG00000019186	*CYP24A1*	1591	20	52769988	52790512
ENSG00000100218	*RTDR1*	27156	22	23401593	23487208
ENSG00000128266	*GNAZ*	2781	22	23412540	23467224
ENSG00000159256	*MORC3*	23515	21	37692487	37758446
ENSG00000159259	*CHAF1B*	8208	21	37757676	37791313
ENSG00000159261	*CLDN14*	23562	21	37832919	37948867

[a]54 positionally-mapped genes with unique Entrez IDs and gene symbols. [b]Genomic positions (hg19).

Supplementary Table 6: Comparison of genotypic models for rs1716707.

**Model**			**N (cases)**	**N (controls)**
Additive	N (allele)	T	10,898	629,730
	N (allele)	C	2,174	147,286
	Odds ratio (95% CI)	1.17 (1.12-1.23)		
	Z-statistic	6.72		
	P-value	1.71×10^{-11}		
			**N (cases)**	**N (controls)**
Recessive	N (genotype)	TT	4,551	255,261
	N (genotype)	TC + CC	1,985	133,247
	Odds ratio (95% CI)	1.19 (1.13-1.26)		
	Z-statistic	44.0		
	P-value	3.24×10^{-11}		
			**N (cases)**	**N (controls)**
Dominant	N (genotype)	TT + TC	6,347	374,469
	N (genotype)	CC	189	14,039
	Odds ratio (95% CI)	1.26 (1.09-1.46)		
	Z-statistic	3.10		
	P-value	0.0019		
			**N (cases)**	**N (controls)**
Full genotypic	N (genotype)	TT	4,551	255,261
	N (genotype)	TC	1,796	119,208
	N (genotype)	CC	189	14,039
	Z-statistic	45.9		
	P-value	1.06×10^{-10}		
			**N (cases)**	**N (controls)**
Homozygote	N (genotype)	TT	4,551	255,261
	N (genotype)	CC	189	14,039
	Odds ratio (95% CI)	1.32(1.14-1.53)		
	Z-statistic	14		
	P-value	1.63×10^{-4}		
			**N (cases)**	**N (controls)**
Heterozygote	N (genotype)	TT	4,551	255,261
	N (genotype)	TC	1,796	119,208
	Odds ratio (95% CI)	1.18(1.12-1.25)		
	Z-statistic	36		
	P-value	1.95×10^{-9}		

Supplementary Table 7: Genotype-phenotype correlations in cohort of kidney stone formers at rs1170174

	DGKH (rs1170174)		
	AA	AG	GG
Serum			
Calcium (mmol/l)	2.33±0.09 (17)	2.36±0.10 (139)	2.34±0.11 (228)
Parathyroid hormone (pmol/l)	5.46±2.71 (18)	4.84±2.45 (140)	5.41±3.30 (234)
25-hydroxy vitamin D (nmol/l)	42.2±15.5 (14)	55.7±25.8 (123)	53.2±27.9 (189)
Urine			
Male patients			
24hr calcium excretion (mmol)	8.14±1.63 (6)	5.95±4.11 (38)	5.09±3.71 (67)
Female patients			
24hr calcium excretion (mmol)	2.75 (1)	4.44±2.59 (16)	5.20±2.74 (31)
Number stone episodes	3.6±3.7 (18)	3.8±5.2 (146)	3.3±5.3 (256)

Supplementary Table 8: UK Biobank Study Population.

Cohort	Number of Samples	Female (%)	Male (%)	Mean Age (SD)
Kidney stone	6,536	33.3	66.7	67.9 (7.63)
Control	388,508	54.5	45.5	66.8 (8.01)

Supplementary Table 9: Japanese Study Population.

Cohort	Source	Genotyping Platform	Number of Samples	Female (%)	Male (%)	Mean Age (SD)
Kidney stone	BBJ	HumanOmniExpressExome or HumanOmniExpress and HumanExome	5,587	1,333 (23.9)	4,254 (76.1)	53.7 (13.8)
Control	JPHC<br>J-MICC<br>ToMMo	HumanOmniExpressExome	28,870	17,492 (60.6)	11,378 (43.7)	56.3 (10.0)

BBJ: Biobank Japan, JPHC: Japan Public Health Centre-based Prospective Study; J-MICC: Japan Multi-Institutional Collaborative Cohort Study; ToMMo: Tohoku Medial Megabank Organisation

Supplementary Table 10: Genomic inflation in the GWA studies. The genomic inflation factor (λGC) and a value for the λGC adjusted to a sample size of 1000 (λ1000) are given for the UK Biobank GWAS, the Japanese GWAS, and the trans-ethnic meta-analysis.

Study	λ_{GC}	λ_{1000}
UK Biobank	1.096	1.030
BioBank Japan	1.164	1.008
Trans-ethnic meta-analysis	1.125	1.021

Supplementary Table 11: Primers

SNP Genotyping Assays	Supplier	Cataolgue Number	SNP ID
rs17216707	ThermoFisher	4351379	C__33659702_10
rs838717	ThermoFisher	4351379	C___8741812_10
rs1170174	ThermoFisher	4351379	C___1932056_10
**qRT-PCR QuantiTect Primer Assays**			
DGKD	Qiagen	QT00068894	-
CASR	Qiagen	QT02394063	-
PGK1	Qiagen	QT00013776	-
GAPDH	Qiagen	QT00079247	-
TUB1A	Qiagen	QT00037149	-
CDNK1B	Qiagen	QT00998445	-

2 Supplementary Figures

Supplementary Figure 1: Regional plots of all GWAS-associated loci in the UK-Japanese meta-analysis.

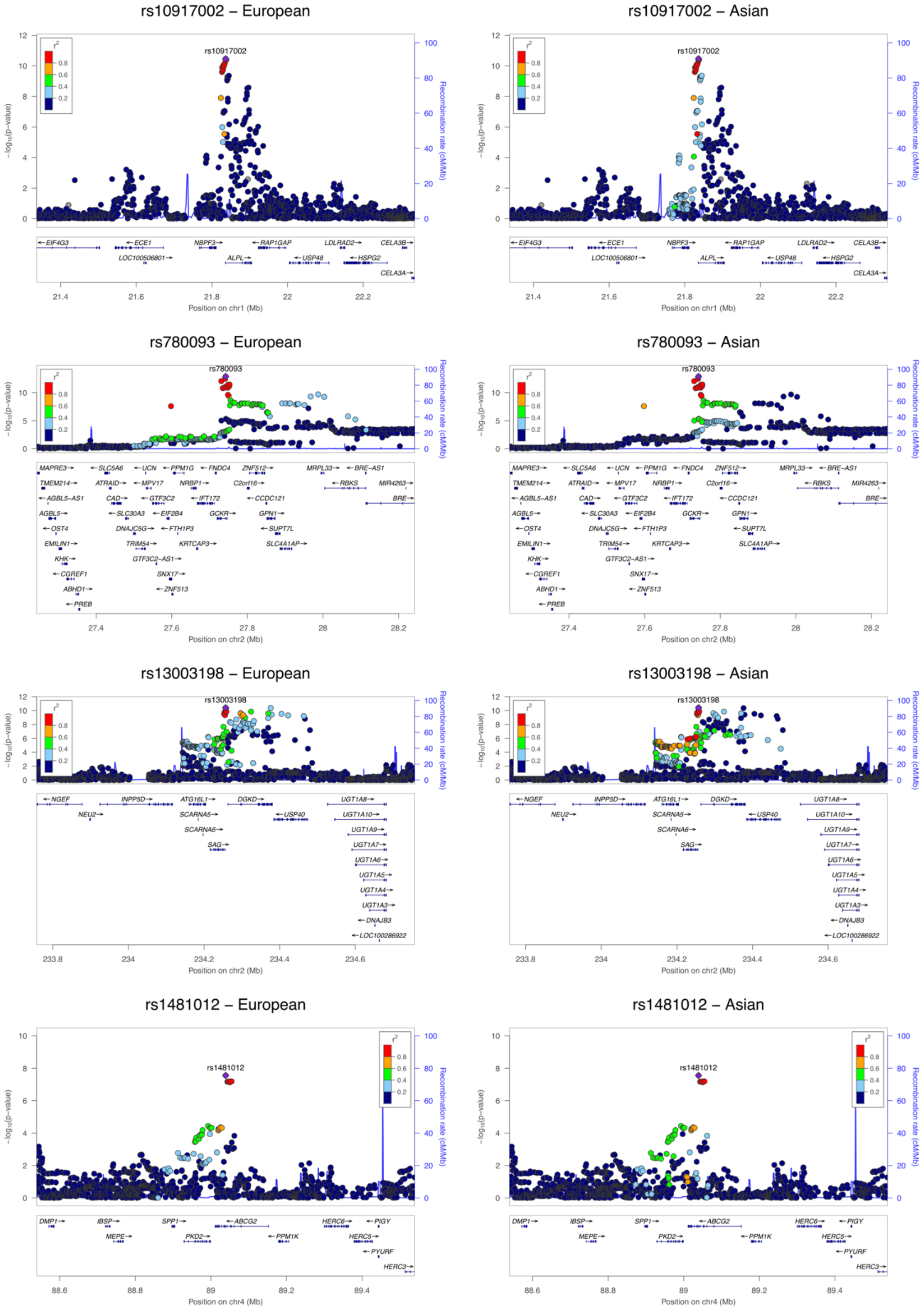

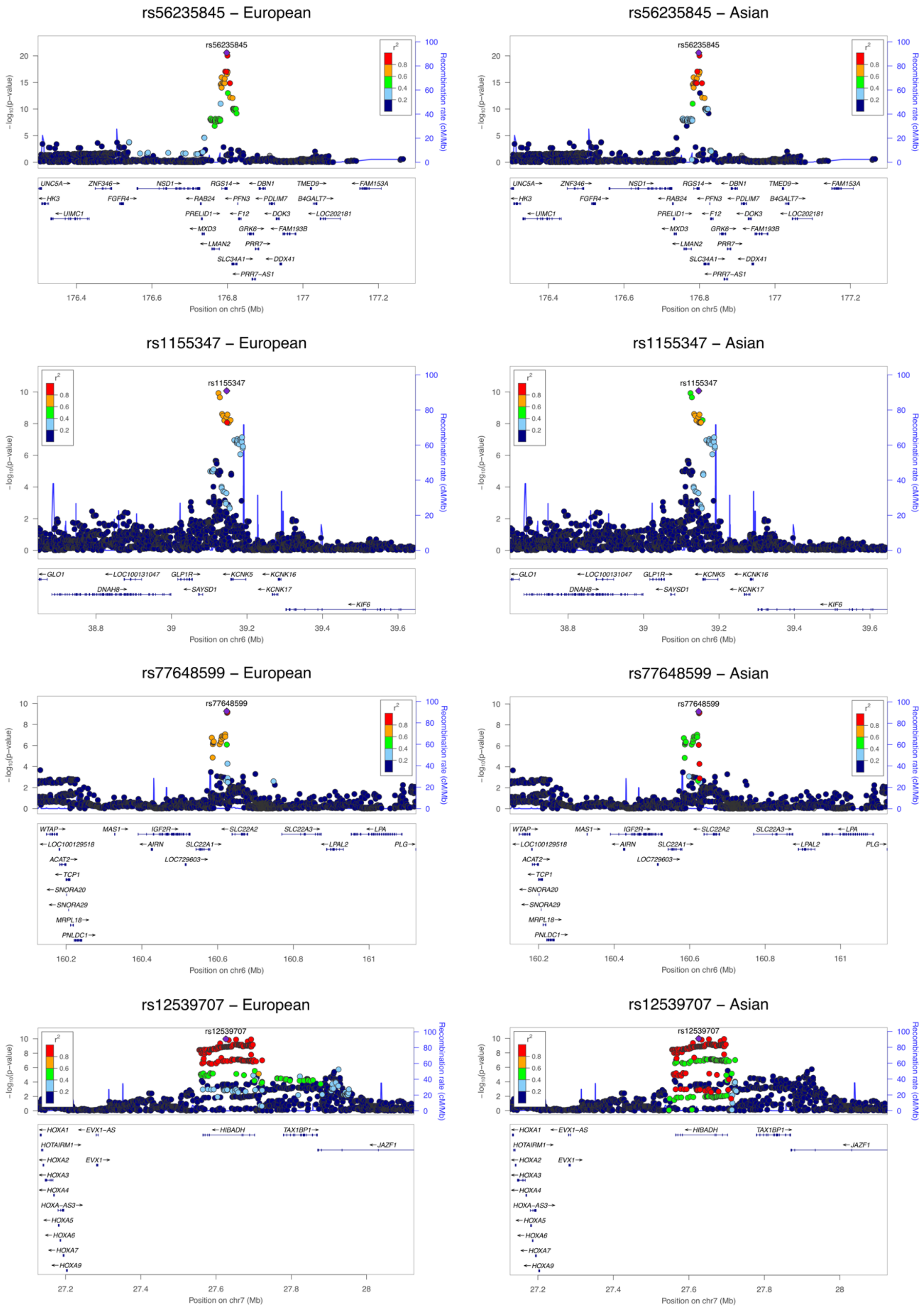
rs56235845 – European
rs56235845 – Asian
rs1155347 – European
rs1155347 – Asian
rs77648599 – European
rs77648599 – Asian
rs12539707 – European
rs12539707 – Asian
Position on chr5 (Mb)
Position on chr6 (Mb)
Position on chr7 (Mb)
Recombination rate (cM/Mb)

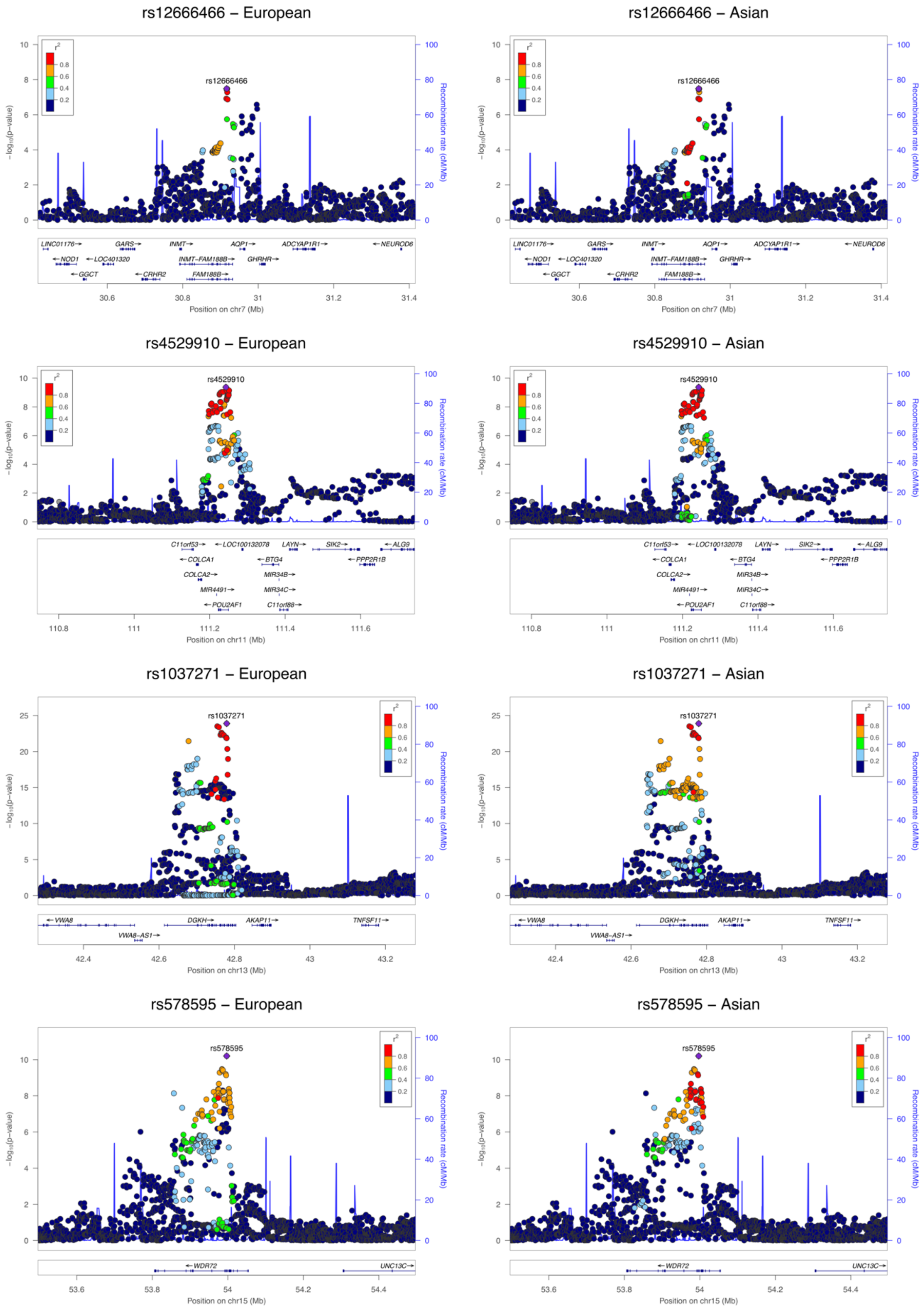
rs12666466 – European
rs12666466 – Asian
rs4529910 – European
rs4529910 – Asian
rs1037271 – European
rs1037271 – Asian
rs578595 – European
rs578595 – Asian
−log10(p−value)
Recombination rate (cM/Mb)
r2
Position on chr7 (Mb)
Position on chr11 (Mb)
Position on chr13 (Mb)
Position on chr15 (Mb)
LINC01176 GARS INMT AQP1 ADCYAP1R1 NEUROD6
NOD1 LOC401320 INMT−FAM188B GHRHR
GGCT CRHR2 FAM188B
C11orf53 LOC100132078 LAYN SIK2 ALG9
COLCA1 BTG4 PPP2R1B
COLCA2 MIR34B
MIR4491 MIR34C
POU2AF1 C11orf88
VWA8 DGKH AKAP11 TNFSF11
VWA8−AS1
WDR72 UNC13C

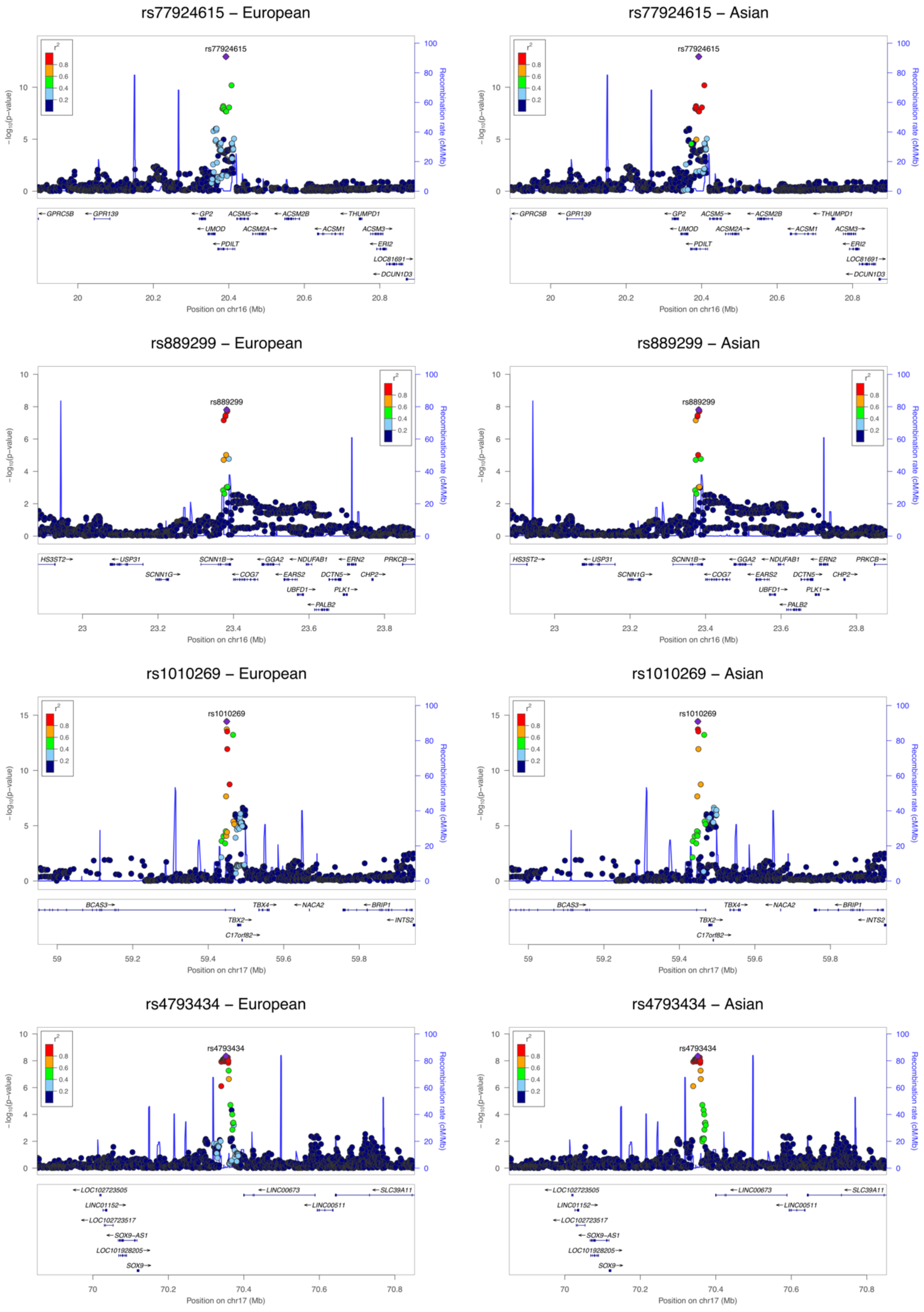
rs77924615 – European
rs77924615 – Asian
rs889299 – European
rs889299 – Asian
rs1010269 – European
rs1010269 – Asian
rs4793434 – European
rs4793434 – Asian
−log10(p−value)
Recombination rate (cM/Mb)
Position on chr16 (Mb)
Position on chr17 (Mb)

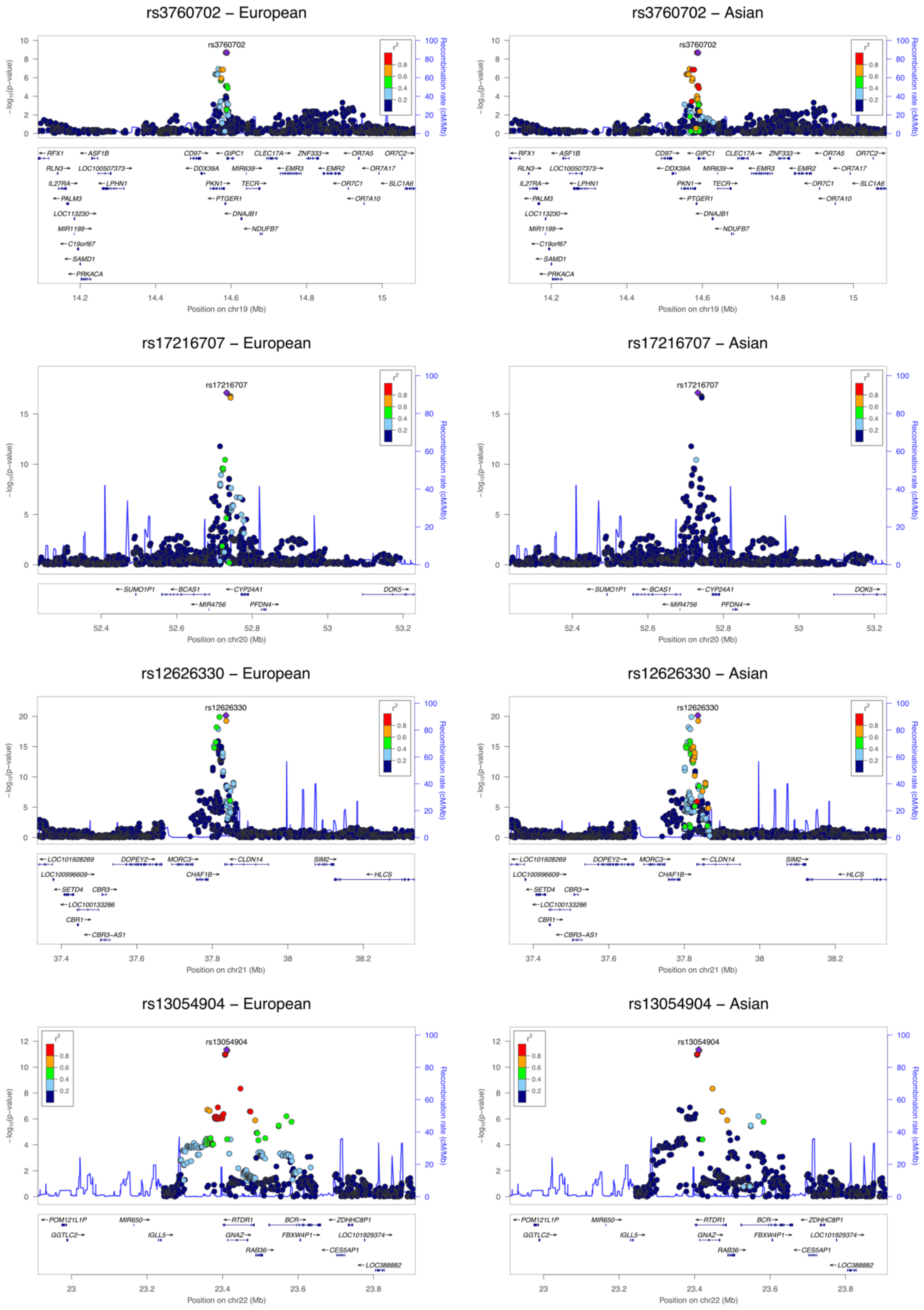
rs3760702 – European
rs3760702 – Asian
rs3760702
−log10(p-value)
Recombination rate (cM/Mb)
r²
RFX1 ASF1B CD97 GIPC1 CLEC17A ZNF333 OR7A5 OR7C2
RLN3 LOC100507373 DDX39A MIR639 EMR3 EMR2 OR7A17
IL27RA LPHN1 PKN1 TECR OR7C1 SLC1A6
PALM3 PTGER1 OR7A10
LOC113230 DNAJB1
MIR1199 NDUFB7
C19orf67
SAMD1
PRKACA
Position on chr19 (Mb)
rs17216707 – European
rs17216707 – Asian
rs17216707
SUMO1P1 BCAS1 CYP24A1 DOK5
MIR4756 PFDN4
Position on chr20 (Mb)
rs12626330 – European
rs12626330 – Asian
rs12626330
LOC101928269 DOPEY2 MORC3 CLDN14 SIM2
LOC100996609 CHAF1B HLCS
SETD4 CBR3
LOC100133286
CBR1
CBR3-AS1
Position on chr21 (Mb)
rs13054904 – European
rs13054904 – Asian
rs13054904
POM121L1P MIR650 RTDR1 BCR ZDHHC8P1
GGTLC2 IGLL5 GNAZ FBXW4P1 LOC101929374
RAB36 CES5AP1
LOC388882
Position on chr22 (Mb)

Supplementary Figure 1. Regional plots of all GWAS-associated loci in the UK-Japanese meta-analysis. LocusZoom plots for the 20 index SNPs are shown, ordered by chromosome number and genomic position. For each SNP, one plot shows the LD relationships in European populations (hg19/1000 Genomes Nov 2014 EUR) and the other in East Asian populations (hg19/1000 Genomes Nov 2014 ASN). SNP position is shown on the x-axis, and strength of association on the y-axis. Genes within 500 kb of the index SNP are shown in the lower panel.

Supplementary Figure 2: Gene-property analysis in MAGMA.

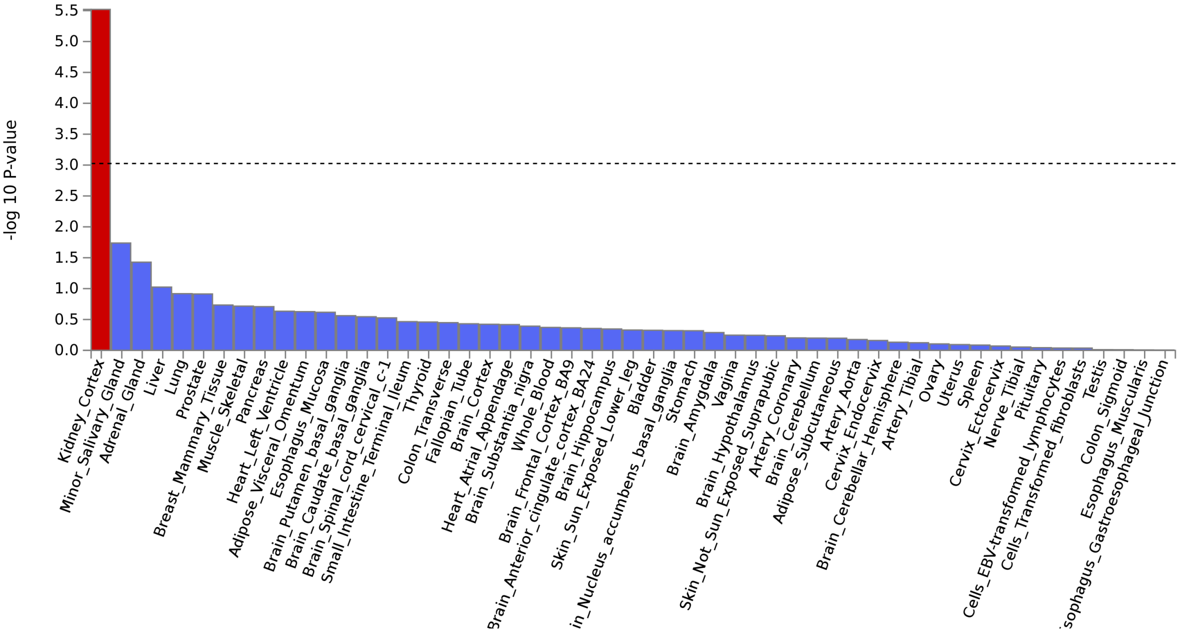

Supplementary Figure 2: Gene-property analysis in MAGMA. MAGMA Tissue Expression Analysis of GWAS-summary data, implemented in FUMA. This tests the relationship between highly expressed genes in a specific tissue and the genetic associations from the GWAS. Gene-property analysis is performed using average expression of genes per tissue type as a gene covariate. Gene expression values are log2 transformed average RPKM (Read Per Kilobase Per Million) per tissue type after winsorization at 50, and are based on GTEx v6 RNA-Seq data across 53 specific tissue types. The dotted line indicates the Bonferroni-corrected α level, and the tissues that meet this significance threshold are highlighted in red.

Supplementary Figure 3: Gene-based enrichment analysis in FUMA.

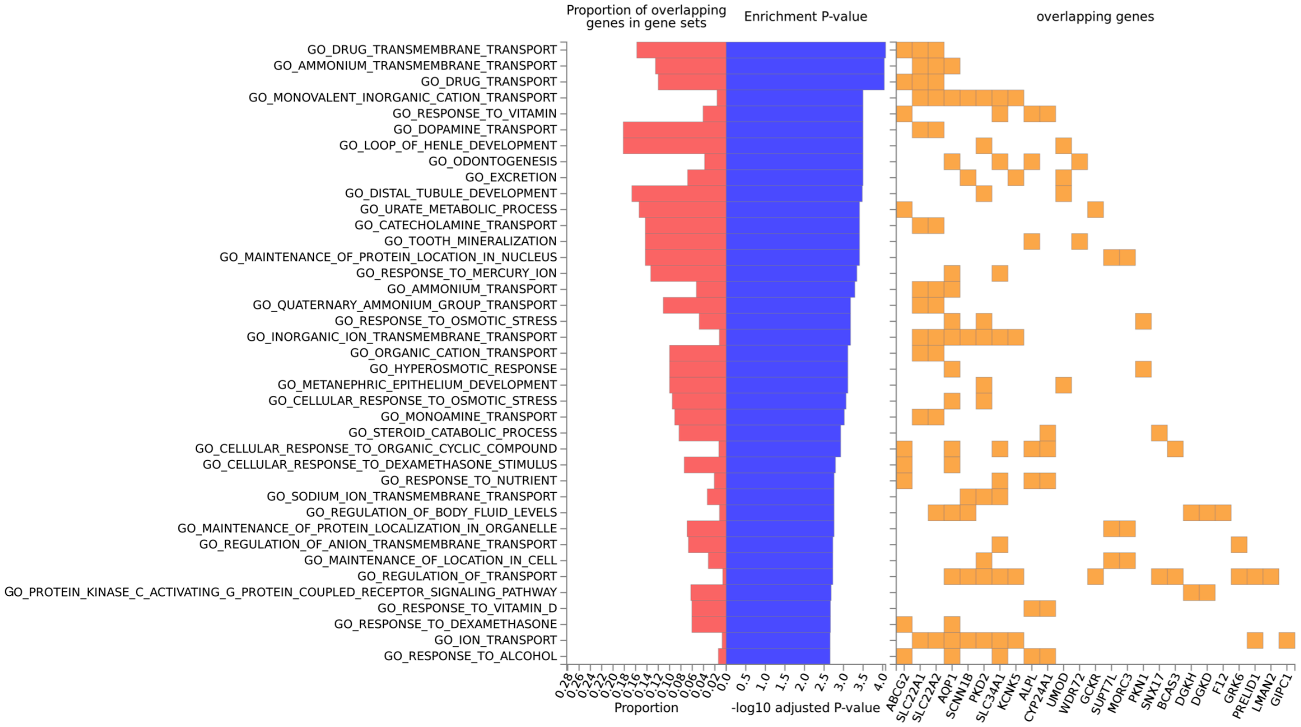

Supplementary Figure 3: Gene-based enrichment analysis in FUMA. This analysis was performed using the GENE2FUNC tool in FUMA, using 54 positionally mapped genes with a unique entrez ID and gene symbol. An adjusted enrichment p-value cut-off of p<0.0025 was used. Gene ontologies are ranked by descending enrichment p-value.

Supplementary Figure 4: Intake of medications in validation cohort participants shown across genotypes.

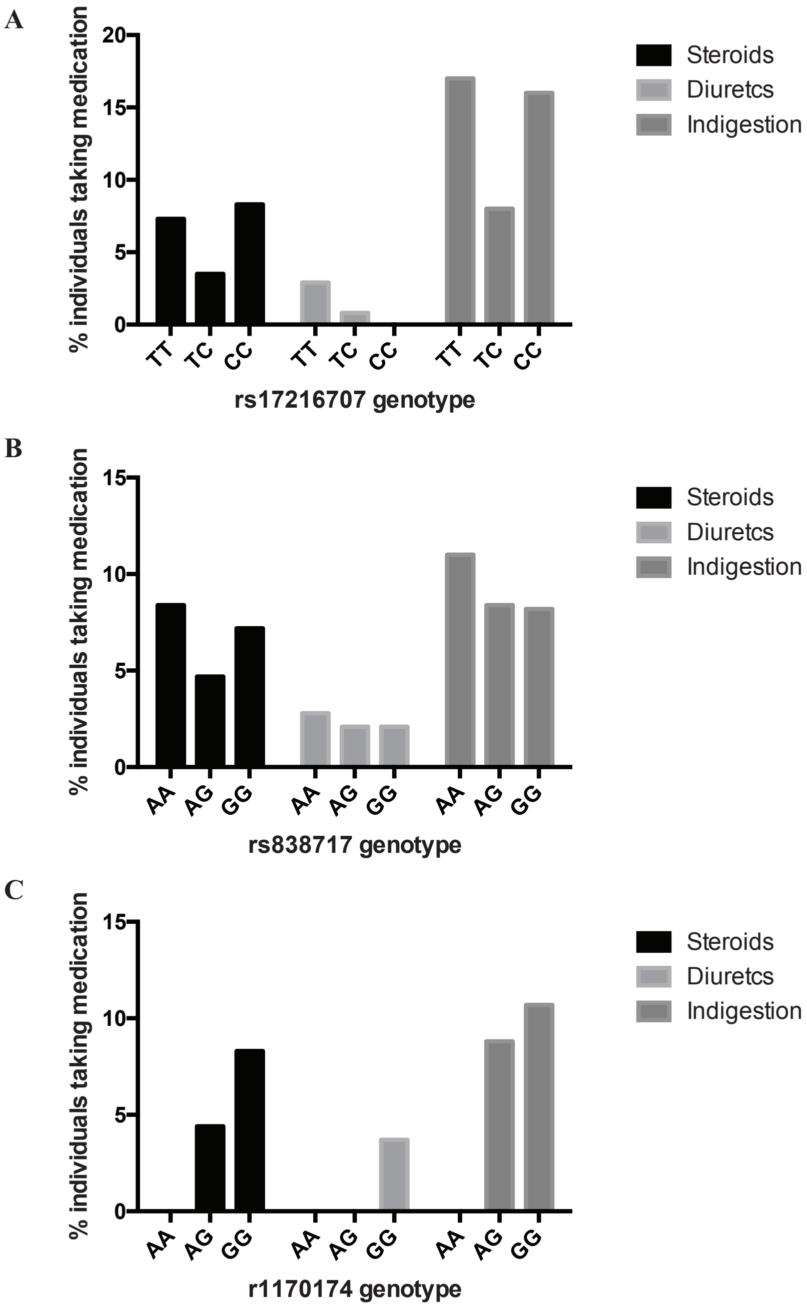

Supplementary Figure 4: Intake of medications in validation cohort participants shown across genotypes (oral steroids, diuretics, and medications for indigestion).

Supplementary Figure 5: CaSR-mediated responses following DGKD knockdown in HEK-CaSR and HEK-CaSR-NFAT cells.

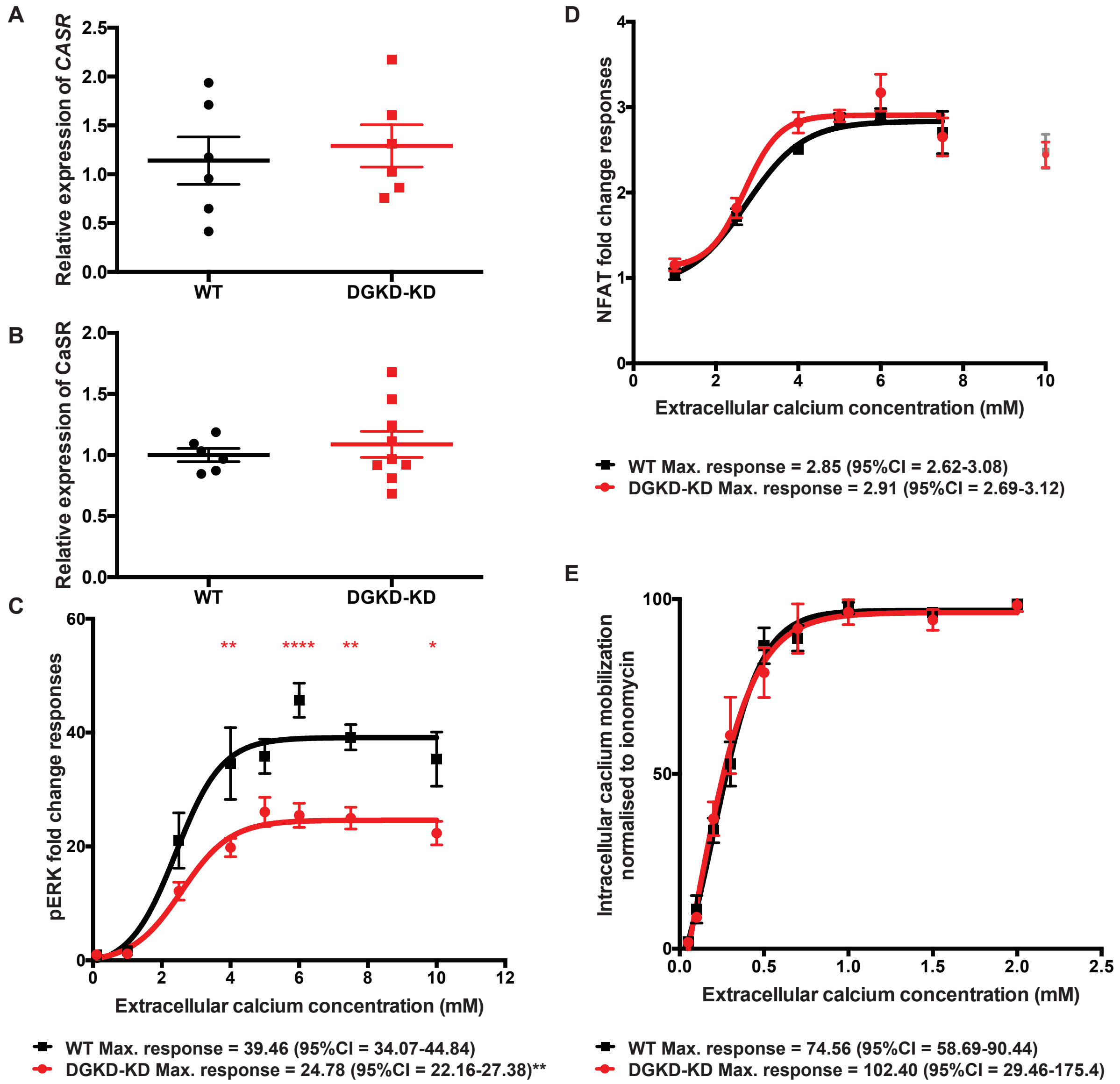

Supplementary Figure 5: CaSR-mediated responses following DGKD knockdown in HEK-CaSR and HEK-CaSR-NFAT cells. Panel A shows relative expression of *CASR*, as assessed by quantitative real-time PCR of HEK-CaSR-SRE cells treated with scrambled (WT) or *DGKD* (DGKD-KD) siRNA. Samples were normalized to a geometric mean of four housekeeper genes: *PGK1*, *GAPDH*, *TUB1A*, *CDNK1B*. n=6 biologically independent

transfections. Panel B shows the relative expression of CaSR, as assessed by densitometry of western blots from cells treated with scrambled (WT) or *DGKD* (DGKD-KD) siRNA. Samples were normalized to α−Tubulin. n=6 biologically independent transfections for WT and n=9 biologically independent transfections for DGKD-KD. Panel C shows pERK responses of HEK-CaSR cells in response to changes in extracellular calcium concentration. Cells were treated with scrambled (WT) or *DGKD* (DGKD-KD) siRNA. The responses ± SEM are shown for 4 biologically biologically independent transfections for WT and DGKD-KD cells. Treatment with *DGKD* siRNA led to a reduction in maximal response (red line) compared to cells treated with scrambled siRNA (black line). Panel D shows NFAT responses of HEK-CaSR-NFAT cells in response to changes in extracellular calcium concentration. Cells were treated with scrambled (WT) or *DGKD* (DGKD-KD) siRNA. The responses are shown for n=5 biologically independent transfections for WT and DGKD-KD cells. Treatment with *DGKD* siRNA did not affect the maximal response (red line) compared to cells treated with scrambled siRNA (black line). Post desensitization points were not included in the analysis (grey, and light red). Panel E shows intracellular calcium responses of HEK-CaSR cells in response to changes in extracellular calcium concentration. Cells were treated with scrambled (WT) or *DGKD* (DGKD-KD) siRNA. Responses were normalized to those elicited by stimulation with ionomycin, and subsequently, to the maximum response observed for WT cells. Responses are shown for n=4 biologically independent transfections for WT and DGKD-KD cells. Treatment with *DGKD* siRNA did not affect the maximal response (red line) compared to cells treated with scrambled siRNA (black line). Statistical comparisons of maximal response were undertaken using F test. Student's t-tests were used to compare relative expression. Two-way ANOVA was used to compare points on dose response curve with reference to WT. Data are shown as mean ±SEM with *$p<0.05$, **$p<0.01$, ****$p<0.0001$. Source data are provided as a Source Data file.
