# Supplementary material for: Genetic variants of calcium and vitamin D metabolism in kidney stone disease
Source: Nat Commun. 2019 Nov 15;10:5175. doi: 10.1038/s41467-019-13145-x (PMC6858460; doi:10.1038/s41467-019-13145-x)
Supplement: Supplementary file 2 — Description of Additional Supplementary Files [file 41467_2019_13145_MOESM2_ESM.pdf]

Title: Supplementary Data 1
Description: Genome-wide significant SNPs in transethnic meta-analysis with likely functional consequences.

Title: Supplementary Data 2
Description: eQTL data for index SNPs at 20 genome-wide significant loci. Source: GTEx v8.
