# Supplementary material for: Genetic variants of calcium and vitamin D metabolism in kidney stone disease
Source: Nat Commun. 2019 Nov 15;10:5175. doi: 10.1038/s41467-019-13145-x (PMC6858460; doi:10.1038/s41467-019-13145-x)
Supplement: Supplementary file 6 — Reporting Summary [file 41467_2019_13145_MOESM6_ESM.pdf]

nature research

Corresponding author(s): Sarah Howles

Last updated by author(s): Sep 6, 2019

# Reporting Summary

Nature Research wishes to improve the reproducibility of the work that we publish. This form provides structure for consistency and transparency in reporting. For further information on Nature Research policies, see Authors & Referees and the Editorial Policy Checklist .

## Statistics

For all statistical analyses, confirm that the following items are present in the figure legend, table legend, main text, or Methods section.

| n/a | Confirmed | |
|---|---|---|
| ☐ | ☒ | The exact sample size (*n*) for each experimental group/condition, given as a discrete number and unit of measurement |
| ☒ | ☐ | A statement on whether measurements were taken from distinct samples or whether the same sample was measured repeatedly |
| ☐ | ☒ | The statistical test(s) used AND whether they are one- or two-sided<br>*Only common tests should be described solely by name; describe more complex techniques in the Methods section.* |
| ☐ | ☒ | A description of all covariates tested |
| ☒ | ☐ | A description of any assumptions or corrections, such as tests of normality and adjustment for multiple comparisons |
| ☐ | ☒ | A full description of the statistical parameters including central tendency (e.g. means) or other basic estimates (e.g. regression coefficient) AND variation (e.g. standard deviation) or associated estimates of uncertainty (e.g. confidence intervals) |
| ☐ | ☒ | For null hypothesis testing, the test statistic (e.g. *F*, *t*, *r*) with confidence intervals, effect sizes, degrees of freedom and *P* value noted<br>*Give P values as exact values whenever suitable.* |
| ☒ | ☐ | For Bayesian analysis, information on the choice of priors and Markov chain Monte Carlo settings |
| ☒ | ☐ | For hierarchical and complex designs, identification of the appropriate level for tests and full reporting of outcomes |
| ☒ | ☐ | Estimates of effect sizes (e.g. Cohen's *d*, Pearson's *r*), indicating how they were calculated |

*Our web collection on statistics for biologists contains articles on many of the points above.*

## Software and code

Policy information about availability of computer code

| | |
|---|---|
| Data collection | N/A |
| Data analysis | PLINK v1.9 and R v3.3.1 for QC of UK Biobank data, and BOLT-LMM v2.3 for association analysis . Trans-ethnic meta-analysis was performed using GWAMA v2.2.2. FUMA v1.3.3 and MAGMA v1.06 were used for in silico analyses. Genotype dosages for population attributable risk were copmuted using QCTOOl v2. |

For manuscripts utilizing custom algorithms or software that are central to the research but not yet described in published literature, software must be made available to editors/reviewers. We strongly encourage code deposition in a community repository (e.g. GitHub). See the Nature Research guidelines for submitting code & software for further information.

## Data

Policy information about availability of data

All manuscripts must include a data availability statement. This statement should provide the following information, where applicable:

- Accession codes, unique identifiers, or web links for publicly available datasets
- A list of figures that have associated raw data
- A description of any restrictions on data availability

Full UK Biobank data is available by direct application to UK Biobank, and summary statistics for the transethnic meta-analysis can be obtained by direct request to the corresponding author.

# Field-specific reporting

Please select the one below that is the best fit for your research. If you are not sure, read the appropriate sections before making your selection.

# Life sciences study design

All studies must disclose on these points even when the disclosure is negative.

| | |
|---|---|
| Sample size | No sample-size calculations were performed. |
| Data exclusions | Our QC methodology detailing sample exclusions is described in the article. |
| Replication | Replicate experiments were successful. |
| Randomization | There was no randomization. |
| Blinding | Blinding was not relevant to this study. |

# Reporting for specific materials, systems and methods

We require information from authors about some types of materials, experimental systems and methods used in many studies. Here, indicate whether each material, system or method listed is relevant to your study. If you are not sure if a list item applies to your research, read the appropriate section before selecting a response.

## Materials & experimental systems

| n/a | Involved in the study | |
|---|---|---|
| ☐ | ☒ | Antibodies |
| ☐ | ☒ | Eukaryotic cell lines |
| ☒ | ☐ | Palaeontology |
| ☒ | ☐ | Animals and other organisms |
| ☒ | ☐ | Human research participants |
| ☐ | ☒ | Clinical data |

## Methods

| n/a | Involved in the study | |
|---|---|---|
| ☒ | ☐ | ChIP-seq |
| ☒ | ☐ | Flow cytometry |
| ☒ | ☐ | MRI-based neuroimaging |

## Antibodies

| | |
|---|---|
| Antibodies used | Anti-CaSR antibody: ADD ab19247, Abcam. Anti a-tubulin antibody T5168; Sigma. Anti-DGKD antibody: SAB1300472, Sigma. |
| Validation | ADD has been extensively used to detect CaSR, including 29 references noted https://www.abcam.com/calcium-sensing-receptor-antibody-5c10-add-ab19347-references.html#active-tab. The anti-a-Tubulin antibody T5168 by Sigma has been used in over 1500 peer-reviewed papers, see https://www.sigmaaldrich.com/catalog/product/sigma/t5168?lang=en&region=GB.<br>DGKD antibodies have minimal reports in the literature. The band noted in our study was smaller than predicted but in line with that on the manufacturers website (https://www.sigmaaldrich.com/catalog/product/sigma/sab1300472?lang=en&region=GB) and demonstrated knockdown in line with qPCR results in our hands. |

## Eukaryotic cell lines

Policy information about cell lines

| | |
|---|---|
| Cell line source(s) | ATCC |
| Authentication | Not authenticated |
| Mycoplasma contamination | Not tested for mycoplasma |
| Commonly misidentified lines<br>(See ICLAC register) | N/A |

## Clinical data

Policy information about clinical studies

All manuscripts should comply with the ICMJE guidelines for publication of clinical research and a completed CONSORT checklist must be included with all submissions.

| | |
|---|---|
| Clinical trial registration | This study was not a clinical trial. UK Biobank has approval from the North West Multi-Centre Research Ethics Committee (11/NW/0382), and this study ("Epidemiology of Kidney Stone Disease") has UK Biobank study ID 885. Ethical committees at each Japanese institute approved the project. Collection of clinical data and biological samples from kidney stone patients attending the Oxford University Hospitals NHS Foundation Trust was approved under the Oxford Radcliffe Biobank research tissue bank ethics (09/H0606/5+5). All patients provided written informed consent. |
| Study protocol | N/A |
| Data collection | N/A |
| Outcomes | N/A |
